# Overcoming Challenging Vascular Anatomy in Chronic Subdural Hematoma: Direct Carotid Bulb Access and Contralateral Middle Meningeal Artery Embolization

**DOI:** 10.1055/a-2603-9286

**Published:** 2025-05-27

**Authors:** Saarang Patel, Zachary Hoglund, Chandrasekhar Palepu, Kyle W. Scott, Visish M. Srinivasan

**Affiliations:** 1 Department of Neurosurgery, Perelman School of Medicine, University of Pennsylvania, Philadelphia, Pennsylvania, United States; 2 Department of Neurosurgery, School of Medicine, St. George's University, Grenada, West Indies

**Keywords:** neurosurgery, vascular, hematoma, carotid bulb access, middle meningeal artery embolization

## Abstract

**Background:**

Middle meningeal artery (MMA) embolization is an emerging intervention for subdural hemorrhage. Few cases discuss the utility of contralateral MMA embolization due to challenging ipsilateral MMA anatomy for this indication.

**Case Presentation:**

A 90-year-old male presented after 6 days of slurred speech and severe headache. A head computed tomography (CT) revealed a left-sided 13-mm subdural hemorrhage, and neck CT angiography revealed left internal carotid artery stenosis at 50%. The carotid stenosis was treated with a standard carotid endarterectomy at the carotid bulb. Despite direct catheterization of the external carotid artery, selective catheterization of the MMA was not feasible. Instead, coils were placed in the left internal maxillary artery spanning the left MMA origin, and the right MMA was selectively embolized using a standard transradial approach. Postoperative CT showed a reduction in subdural hematoma (SDH) size, and the patient was discharged in stable condition on postoperative day 6.

**Conclusion:**

This case presents a rescue or salvage maneuver for MMA embolization for SDH with a favorable safety profile and outcome.

## Introduction

Chronic subdural hematoma (cSDH) is one of the most common neurological conditions. It is associated with aging and is projected to greatly increase in prevalence. 1 Current methods of treating subdural hematoma (SDH) have significant rates of complications and recurrence. 1 2 Middle meningeal artery (MMA) embolization is an emerging treatment for cSDH. It involves occlusion of vessels and neovasculature that supply the hematoma in an effort to mitigate the growth of the hematoma and increase its resorption. 3 4 5 In this way, it is hypothesized to directly target the underlying mechanism of cSDH. Many studies have shown its effectiveness with clinical trials having been completed, but not yet published. 6 7 8 9 10 11 12 13 Careful patient selection has been emphasized in many of the studies evaluating MMA embolization. The feasibility of this procedure with unfavorable anatomy or rescue methods has not been extensively described in the literature.

The following case involves intraoperative direct carotid access and contralateral MMA embolization when ipsilateral access was not possible. Direct carotid access can have several indications including unfavorable anatomy or aortic dissection. Further, direct carotid access allows the operator to completely bypass unfavorable anatomy, which shortens time to reperfusion. For percutaneous direct access, ultrasound guidance is typically used; despite this, arteriotomy closure can be associated with several complications as well. Such complications can be minimized by using the “cut-down” technique at the cost of longer operative times. 14

Contralateral MMA embolization has been used as adjunctive treatment in recurrent cSDH, typically after unilateral MMA embolization failed to decrease recurrence. 15 Although the contralateral side is embolized, blood flow is decreased to the ipsilateral side via anastomoses. Pathophysiology of cSDH is not completely understood, but evidence suggests that the formation of the membrane surrounding the cSDH follows an inflammatory process that forms fragile neovasculature. This fragile vasculature is hypothesized to be the source of the hemorrhage and exudate seen in cSDH. 16 By embolizing the contralateral MMA, collateral supply to the neovasculature in the inflammatory membrane is interrupted from the contralateral side resulting in increased absorption of the hematoma.

Here, we present a case of a 90-year-old patient in which a rescue or salvage maneuver for MMA embolization was performed for a cSDH.

## Case Presentation

**Fig. 1:**
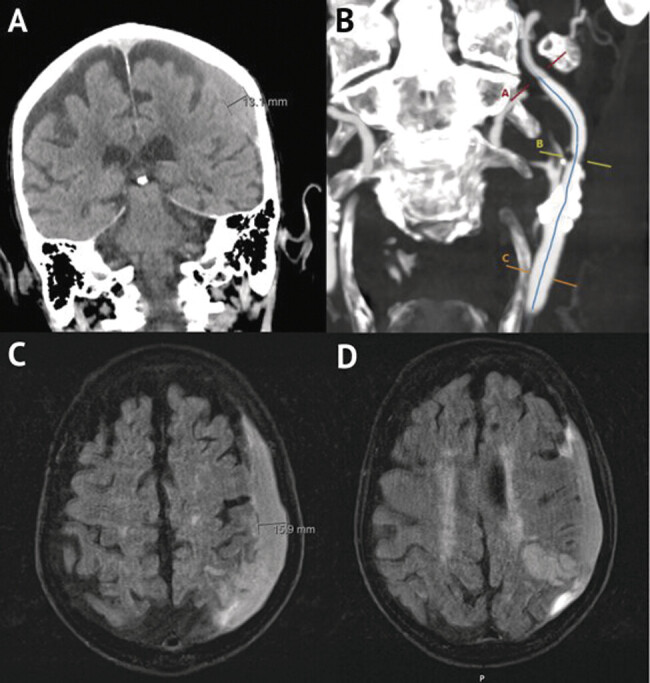
Preoperative and imaging on presentation. ( **A** ) Noncontrast head CT revealing a left SDH 13 mm in thickness. ( **B** ) CT neck angiography with contrast demonstrating internal carotid artery stenosis approximately 50% by the NASCET criteria. ( **C** ) Head MRI taken approximately 8 hours later redemonstrating a left SDH. Thickness increased to 16 mm. ( **D** ) Head MRI demonstrating a subacute infarct in the left parietal lobe with findings suggestive of petechial hemorrhage. CT, computed tomography; MRI, magnetic resonance imaging; SDH, subdural hematoma.

A 90-year-old male with a history of hypertension, hyperlipidemia, stage three chronic kidney disease, dementia, and unsteady gait with frequent falls presented to the emergency department after 6 days of slurred speech and severe headache, as reported by the patient's family. According to the patient's family, the patient had last fallen 2 weeks prior to admission, head strike was unknown. Upon admission, the patient was positive for speech difficulty. A head computed tomography (CT) revealed a 13-mm left SDH with 5-mm midline shift, left frontal infarct, and a subacute left parietal infarct in the small branch territory of the left middle cerebral artery ( Fig. 1A ). Neck CT angiography was significant for left carotid stenosis greater than 50% by the NASCET criteria ( Fig. 1B ). Therefore, the patient was referred to neurosurgery for management of both the symptomatic carotid stenosis and the SDH.

A magnetic resonance imaging taken approximately 8 hours later confirmed the left parietal infarct with petechial hemorrhage and redemonstrated the left SDH but increased up to 16 mm ( Fig. 1C, D ). Bilateral carotid ultrasonography confirmed 50 to 79% stenosis of the left internal carotid artery (ICA). Neurointensive care unit evaluation calculated a National Institutes of Health Stroke Scale score of 9. The patient was started on levetiracetam, atorvastatin, and, 3 days after admission, aspirin. A decision was made to perform a hybrid open left carotid endarterectomy and endovascular left MMA embolization with attempted direct carotid bulb access to address the SDH and symptomatic carotid stenosis, respectively. The procedure was performed on day 5 of admission.

The procedure was performed under general anesthesia. Ultrasound was used to localize the carotid bifurcation and incision. Standard carotid endarterectomy was performed with the superior thyroid artery being dissected and temporarily clipped.

**Fig. 2:**
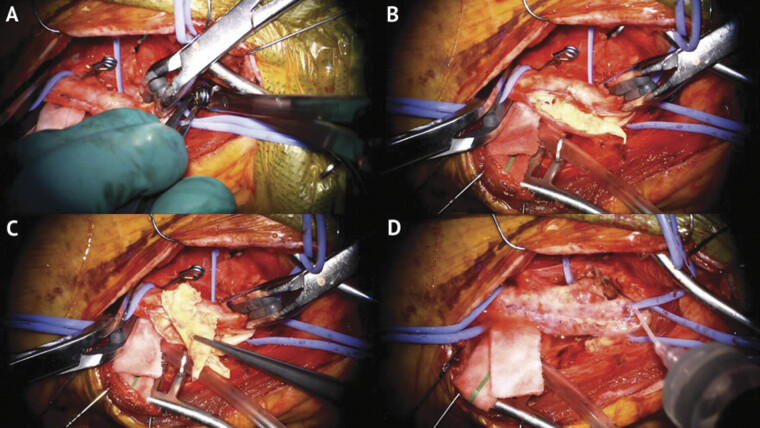
Intraoperative photography of the left carotid endarterectomy. ( **A** ) Placement of aneurysm clips on the ICA, ECA, and CCA. ( **B** ) Open carotid bulb demonstrating significant plaque. ( **C** ) Removal of carotid plaque. ( **D** ) Closed carotid bulb prior to attempted catheter access for MMA embolization. CCA, common carotid artery; ECA, external carotid artery; ICA, internal carotid artery; MMA, middle meningeal artery.

An operating microscope was used for microdissection, and Fig. 2 shows intraoperative photographs taken with the microscope. Heparin (5,000 units) was administered, and the ICA was isolated. The mean arterial pressure was increased by 20%, and aneurysm clips were applied to the ICA, ECA, and common carotid artery (CCA). The artery was opened, and the plaque was removed circumferentially with Potts scissors. The artery was “feathered” for smooth contours, irrigated with heparinized solution, and closed with 7–0 Prolene sutures. The suture line was back-bled and reirrigated before final closure.

**Fig. 3:**
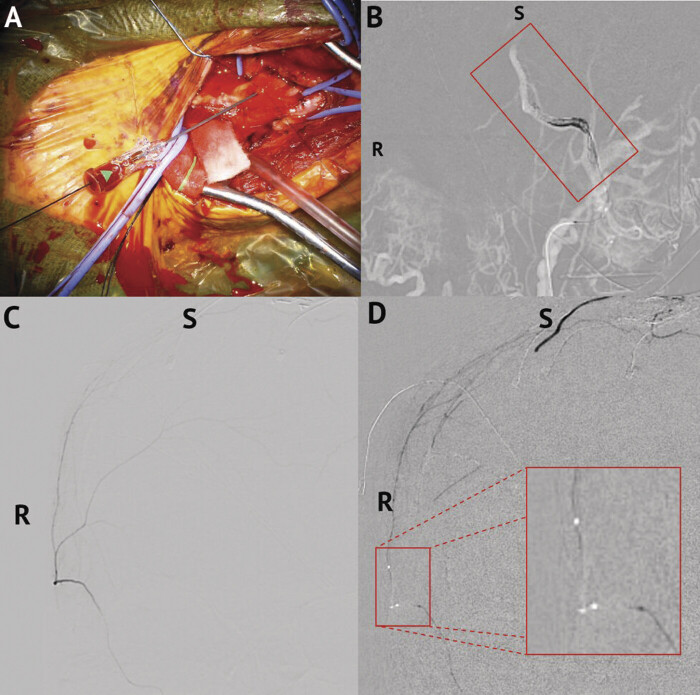
MMA embolization. ( **A** ) Attempted direct carotid bulb access. Cannulation of carotid bulb using a micropuncture needle to introduce a 4-Fr introducer sheath. ( **B** ) Coiling of left IMAX artery. ( **C** ) Selective angiography of the right MMA before embolization. ( **D** ) Embolization of the right MMA. MMA, middle meningeal artery.

As part of the hybrid procedure, a U-stitch was placed around the proximal ECA. The ECA was cannulated with a micropuncture needle, and a 4-French (Fr) introducer sheath was advanced for MMA embolization ( Fig. 3A ).

The sheath terminated in the distal ECA, with limited flow. Angiography through the sheath showed very poor visualization of the left MMA origin, which precluded selective embolization. The ostium was likely small and downregulated due to the chronic ECA origin stenosis and was atherosclerotic as well. Thus, an “indirect” embolization was performed by coil embolization of the left internal maxillary artery (IMAX) spanning the MMA origin ( Fig. 3B ).

A decision was made to pursue right MMA embolization in an attempt to directly cast the membrane via MMA collaterals. A right radial artery sheath was then placed using standard technique. A 6-Fr Benchmark (Penumbra Inc., Alameda, California, United States) over a Sim Select and Glidewire (Terumo Interventional Systems, Somerset, New Jersey, United States) were used to select the right CCA to access the right MMA. Using a microsystem of Excelsior SL 10 (Stryker Corp., Kalamazoo, Michigan, United States) over a Synchro2 microwire (Stryker Corp., Kalamazoo, Michigan, United States), the right MMA was selected and a selective angiography was performed ( Fig. 3C ). The catheter was subsequently flushed with D5W. A 4:1 mixture of lipiodol and Trufill N-butyl cyanoacrylate (nBCA) glue (Johnson and Johnson MedTech, Irvine, California, United States) was used to embolize the right MMA, effectively eliminating left MMA circulation through collateralization of the left and right MMA ( Fig. 3D ). The ECA U-stitch was tied after sheath removal, ensuring hemostasis.

Final angiography of the right external carotid and common carotid arteries performed through the Benchmark catheter confirmed successful embolization, and the radial sheath was removed.

**Fig. 4:**
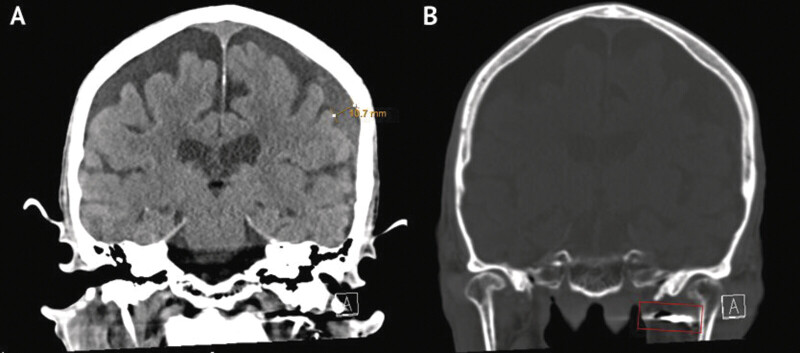
Postoperative head CT. ( **A** ) Redemonstrated left subdural hematoma reduced to 11 mm. ( **B** ) Coils placed in left IMAX demonstrated on CT. CT, computed tomography.

There were no intraoperative complications. The patient received a follow-up head CT on postoperative day 6 demonstrating no midline shift, the SDH reduced in size to 11 mm, the subacute infarct was stable, and there was no new hemorrhage ( Fig. 4 ). The patient was discharged home on postoperative day 6 in stable condition and continued on aspirin and atorvastatin. At discharge, the patient was awake and following simple commands with normal language and mild dysarthria.

## Discussion

Herein, we presented a rescue or salvage maneuver for MMA embolization for an SDH with a favorable safety profile and outcome in a 90-year-old male. Clinical trials have been underway to better examine the benefits of MMA embolization for cSDH. To date, there are five clinical trials studying MMA embolization for cSDH. 9 10 11 12 13

### Direct Carotid Access

Direct carotid access can have multiple indications. Tortuosity of the aortic arch and carotid artery or anatomic anomalies of the brachiocephalic trunk can prevent endovascular navigation. Type A aortic dissection can make endovascular navigation hazardous and time-consuming. Strokes may develop immediately after aortic dissection repair. A hybrid suite allows for rapid transition between surgical and angiographic procedures, although percutaneous technique can be used in standard angiography suites. This decreases the risk of postoperative complications via reduction in surgical time. 14 15

In our case, the indication was carotid stenosis. The hybrid endarterectomy allowed both for clearing of the plaque and access to the ECA via the endarterectomy conduit. Unfortunately, MMA selection was not possible due to anatomic constraints, but this approach may be viable in other patients.

Direct carotid puncture can be associated with certain serious complications such as pseudoaneurysm, dissection, and neck hematoma formation, but several cases have been described with good outcomes. 17 18 19 20 21 22 23 24 25 “Cut-down” methods for carotid access have been described with decreased complications despite increased operative time. 18 19 23 24 Case series and studies have shown some early evidence for more favorable outcomes with the cut-down technique, mainly with the formation of cervical hematoma. Risk of hematoma formation has been speculated to relate to sheath size. 24 In our case, puncture of the ECA was done with a micropuncture needle and a 4-Fr introducer. Direct exposure also provides a wider field for puncture. This allows for repeat punctures while maintaining hemostasis. 18 Percutaneous and direct exposure methods have not yet been extensively studied mainly since they are alternative methods used when the primary methods are not feasible. A retrospective analysis of 548 patients undergoing endovascular management for strokes showed only 8 (1.46%) required transcervical exposure. 18

### Contralateral Middle Meningeal Artery Embolization

Contralateral MMA embolization alone has been previously described in one case study. A patient presented with a tortuous MMA at its origin, preventing distal catheterization. Proximal coil occlusion was performed, which did not result in resolution. The contralateral MMA was embolized, leading to near-complete resolution at 3 months. 26 There is some further evidence of the interactions between the ipsilateral and contralateral vessels in cSDH pathology. Neovascularization on the contralateral side of an SDH has been observed. 27 Patients who underwent contralateral MMA embolization for bilateral cSDH saw a decrease in cSDH diameter and no recurrence. 28 A patient with a fractured left skull over the left MMA presented with SDH on the right. On CT, the left MMA was noted to be dilated and was embolized in addition to evacuation and craniotomy of the right side. 29 A patient with a recurring cSDH after unilateral MMA embolization presented with enlarged anastomoses feeding from the contralateral MMA. Embolization of the contralateral side resolved the SDH at 6 months. 15 Penetration of liquid embolic, deployed in the ipsilateral MMA, to the contralateral MMA, was observed in 28% of cases in a retrospective study of 89 patients. 30 cSDH is driven by inflammatory processes, which may be brain wide. 31 These findings suggest a greater interplay of the right and left MMAs in cSDH pathology and suggest a more brain-wide phenomenon not restricted to the initial bleeding site.

## Conclusion

While MMA embolization is an established technique for recurrent cSDH with increasing evidence for initial or prophylactic treatment, our case demonstrates a unique approach: attempting direct access through the carotid bulb and contralateral MMA embolization. Despite accessing the carotid bulb intraoperatively, the MMA was unnavigable due to atherosclerosis, which resulted in direct-access IMAX coiling and contralateral MMA access and embolization. The resulting stabilization of the hematoma shows the viability of this as a rescue or salvage maneuver with a favorable safety profile and outcome. Additionally, it suggests that cSDH interventions that utilize bilateral MMA embolization are an effective approach to mitigate bleeding by eliminating blood flow from collateral circulation with the contralateral MMA in addition to ipsilateral flow.
